# Clinical value of assessing motor performance in postacute stroke patients

**DOI:** 10.1186/s12984-021-00898-0

**Published:** 2021-06-24

**Authors:** D. Flury, F. Massé, A. Paraschiv-Ionescu, K. Aminian, A. R. Luft, R. Gonzenbach

**Affiliations:** 1 Faculty of Medicine, University of Zurich, Zurich, Switzerland; 2 Laboratory of Movement Analysis and Measurement, Ecole Polytechnique Fédérale de Lausanne, Lausanne, Switzerland; 3 Department of Neurology, University Hospital of Zurich, Zurich, Switzerland; 4 Center for Neurology and Rehabilitation, Cereneo, Vitznau, Switzerland

**Keywords:** Stroke, Rehabilitation, Wearable inertial sensors, Motor performance

## Abstract

**Background:**

Rehabilitative treatment plans after stroke are based on clinical examinations of functional capacity and patient-reported outcomes. Objective information about daily life performance is usually not available, but it may improve therapy personalization.

**Objective:**

To show that sensor-derived information about daily life performance is clinically valuable for counseling and the planning of rehabilitation programs for individual stroke patients who live at home. Performance information is clinically valuable if it can be used as a decision aid for the therapeutic management or counseling of individual patients.

**Methods:**

This was an observational, cross-sectional case series including 15 ambulatory stroke patients. Motor performance in daily life was assessed with body-worn inertial sensors attached to the wrists, shanks and trunk that estimated basic physical activity and various measures of walking and arm activity in daily life. Stroke severity, motor function and activity, and degree of independence were quantified clinically by standard assessments and patient-reported outcomes. Motor performance was recorded for an average of 5.03 ± 1.1 h on the same day as the clinical assessment. The clinical value of performance information is explored in a narrative style by considering individual patient performance and capacity information.

**Results:**

The patients were aged 59.9 ± 9.8 years (mean ± SD), were 6.5 ± 7.2 years post stroke, and had a National Institutes of Health Stroke Score of 4.0 ± 2.6. Capacity and performance measures showed high variability. There were substantial discrepancies between performance and capacity measures in some patients.

**Conclusions:**

This case series shows that information about motor performance in daily life can be valuable for tailoring rehabilitative therapy plans and counseling according to the needs of individual stroke patients. Although the short recording time (average of 5.03 h) limited the scope of the conclusions, this study highlights the usefulness of objective measures of daily life performance for the planning of rehabilitative therapies. Further research is required to investigate whether information about performance in daily life leads to improved rehabilitative therapy results.

## Introduction

When a rehabilitation physician meets with a postacute stroke patient for counseling and rehabilitation program planning, decisions are usually based on two types of information: the results of a clinical examination of functional capacity (i.e., what a person can do in a standardized, controlled environment) and the patient’s subjective report on limitations and problems in daily life. With this information, the rehabilitation professional and the patient set specific goals together, with the objective of improving functional performance (i.e., what a person does in his daily life) [1]. An objective measurement of functional performance was not available for a long time, but with the development of wearable sensors, it is now increasingly used in rehabilitation. Wearable sensor technology allows the collection of data that had previously been missing: the ‘objective measurement of clinically important naturalistic behaviors' [2]. Ideally, information about performance would be available for the planning and monitoring of a rehabilitation program and would include several aspects, such as overall physical activity, walking behavior and upper-limb use.

Studies involving wearable sensors generally report low physical activity levels, low walking performance and little use of the affected arm in daily life in stroke patients at the population level [3, 4]. However, the variability of daily life performance measures among patients was considerable in most studies [5, 6]. Demographic or stroke-related variables did not [7] or only partially [6, 8–10] explain the performance variability.

Potential applications of sensor-derived performance measures in rehabilitation programs have been described by many authors [11, 12], but we are not aware of any studies that examined the value of such performance information in individual patients receiving clinical rehabilitation. Additionally, with few exceptions [13, 14], most studies that employed wearable sensors to measure daily life performance in stroke patients focused on either upper or lower limbs. However, the clinical situation of a patient initiating a rehabilitation program would, in most cases, require a comprehensive assessment of upper-limb activity, walking behavior and physical activity.

We hypothesize that comprehensive, sensor-derived performance information is clinically valuable for the planning of rehabilitation programs for individual stroke patients who live at home. Performance data are deemed clinically valuable if they can be used as decision aids for therapeutic management or for counseling in individual patients [15]. We explore the clinical value in a narrative style, with a focus on individual patient performance and capacity data.

Daily life performance was recorded with a series of wearable sensors placed on the upper and lower extremities and the trunk. The wearable sensors were placed on the patient in the clinic by a clinical scientist as suggested by others [16] because the handling and placement of the wearable sensors was judged too complicated to be done independently by the stroke patients. Recordings were initiated during a routine medical consultation in the morning and lasted until late afternoon of the same day. We intended to measure performance under a scenario that is feasible in routine clinical practice. Therefore, repeatedly visiting patients over several days to help with sensor handling (e.g., for undoing/redoing or charging of sensor modules) was not an option, considering the time and cost constraints in most healthcare systems. On these grounds, a longer recording period was not an option.

## Methods

### Participants

Fifteen postacute stroke patients (i.e., more than 3 months post stroke) living at home and attending outpatient rehabilitation in the Rehabilitation Center Valens were consecutively recruited between October and December 2014. Patients were included if they were able to walk more than 10 m without supervision. All recruited participant finished the study. The patient characteristics are described in Table 1.

**Table 1:** Population characteristics

Variable	Stroke patients (N = 15)
Age (years)	59.9 ± 9.8 (mean ± SD)
Range	31.9–76.9
Body Mass Index (kg/m^2^)	25.5 ± 3.8 (mean ± SD)
Sex Women Men	6 9
Time post stroke (years)	6.5 ± 7.2 (mean ± SD)
Range	0.33–22.33
Type of Stroke Ischemic Hemorrhagic	8 7
Side of hemiparesis Left Right	11 4
Dominant hand affected	5

### Study design

The present study was carried out as an observational, cross-sectional, case series. The participants were assessed during a routine medical consultation at the Rehabilitation Center Valens in the morning between 9 and 11 am. During the clinical consultation, standardized assessments of functional capacity were performed, and patient-reported measures were collected. At the end of the consultation, the patients were equipped with wearable inertial sensors. Following the consultation, daily life performance was assessed with these sensors when the patients performed their daily routine outside the hospital. The only instruction to the patients was to behave as usual. On the evening of the same day, the sensors were collected at the patient’s home. The potential clinical value of performance data for individual patients is discussed in a narrative style.

### Clinical assessments and patient-reported measures

The National Institutes of Health Stroke Scale (NIHSS) [17] was used to quantitatively measure neurological deficit after stroke. The Fugl-Meyer Assessment (FMA) [18] was used as a stroke-specific impairment index of motor function, impairment, balance and joint functioning. The Motor Activity Log-14 (MAL-14) was used as a patient-reported outcome measure for the amount of use (AOU) of the more-affected arm [19]. The Action Research Arm Test (ARAT) [20] was used to assess upper-limb functioning in activities of daily living. Walking and balance were assessed with the 10-Meter Walk Test (10 MWT) [21], the Timed Up and Go Test (TUG) [22] and the Berg Balance Scale [23]. Independence was quantified with the Modified Rankin Scale (MRS) [24] and the Barthel Index [25] based on the patient’s report.

### Wearable sensor system: acquisition and sensor configuration

The wearable inertial sensor device Physilog®4 (Gait Up Ltd., Lausanne, CH) was used for data collection. Each lightweight device (19 g) is 50 × 37 × 9.2 mm and integrates a microcontroller, 4 GB internal storage, a wireless radio interface, a three-axis accelerometer, a three-axis gyroscope, a three-axis magnetometer, a barometer, and a rechargeable battery (more details about Physilog®4 are available at GaitUp_Datasheet_Physilog_4).

In this study, only the accelerometer and gyroscope signals were processed and acquired at a sampling frequency of 200 Hz to enable further postprocessing by motion-processing algorithms. The wireless radio interface embedded in the device ensured data synchronization using periodic timestamped beacons. Linear interpolation was applied to resynchronize the signals.

**Fig. 1:**
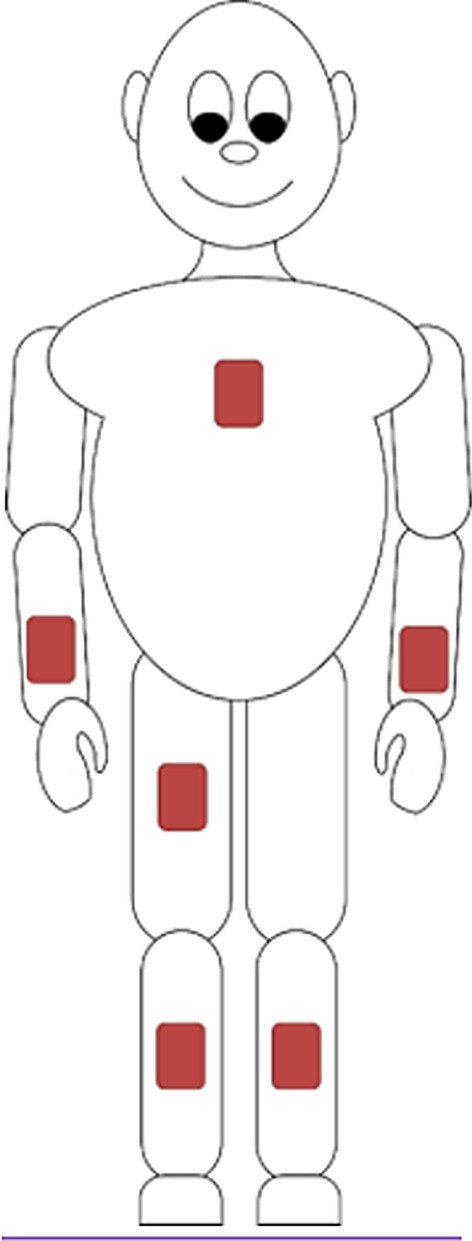
Placement of the wearable sensor devices

The wearable sensors were placed as shown in Fig. 1 to capture the performance parameters of upper-limb activity, walking, and main activities (lying, sitting, standing and walking). For body posture detection, one sensor device was attached to the chest with a medical patch (Medipore® 3 M, Rüschlikon, Switzerland), one on each wrist and each lower leg (shank) using Velcro® straps (Velcro IP Holdings LLC, UK) and one on the impaired lateral thigh using a medical patch (Medipore® 3 M, Rüschlikon, Switzerland). For walking detection and gait analysis, sensors were placed on each lower leg (shank) using Velcro® straps. The monitoring of arm activity was performed using one sensor on each wrist using Velcro® straps.

All measurements started in the late morning and ended in the late afternoon, resulting in an average recording time of 5.03 ± 1.1 h. In one patient (#9), the fixation of a shank sensor loosened during recording. The period when sensor data were lost was excluded from the recording, resulting in a shorter recording time.

### Wearable sensor system: performance metric extraction

Clinically relevant information was extracted and calculated from the accelerometer and gyroscope raw data in MATLAB® (The MathWorks Inc.; Version 7.10.0 (R2010a), Natick, Massachusetts USA) using three existing algorithms: a classifier of main activities of daily living (sitting, walking, standing and lying) [26], a step detection algorithm [27], and an arm-activity detection algorithm [28]. The activity classification algorithm, used in various clinical studies [29], detects the body posture (upright, sitting or lying) using the vertical and frontal acceleration signals from the trunk sensor and the frontal acceleration signal from the thigh sensor. The two detected upright activities, walking and standing, were distinguished using the two leg sensors located on the shanks. The step count (number of steps) was also computed from the gyroscope sensors placed on the shanks [27]. The principal axis of the sensor was aligned in the movement direction using principal component analysis [30]. The algorithm detects the swing and stance phases for each leg by thresholding the principal axis gyroscope information after high-pass filtering (cutoff frequency of 0.25 Hz). A walking episode starts after one full gait cycle, i.e., the occurrence of two consecutive steps, one from the right leg and the other one from the left. The maximum gait cycle time was fixed to 6 s. In the upright posture, the subject was considered walking if they were not standing.

Arm activity data were extracted from the sensors placed on each wrist[28]. After low-pass filtering of the 3-axis gyroscope raw data, the analytical representation of each axis signal was extracted using the Hilbert transform. A binary threshold, adapted to the population and set to 20 deg/s, was then applied on each axis. The arm was considered active if at least one analytical representation exceeded the defined threshold. The arm activity time (in seconds) was the duration of arm activity according to this definition during the recording time.

### Wearable sensor system: postprocessing and normalization

**Fig. 2:**
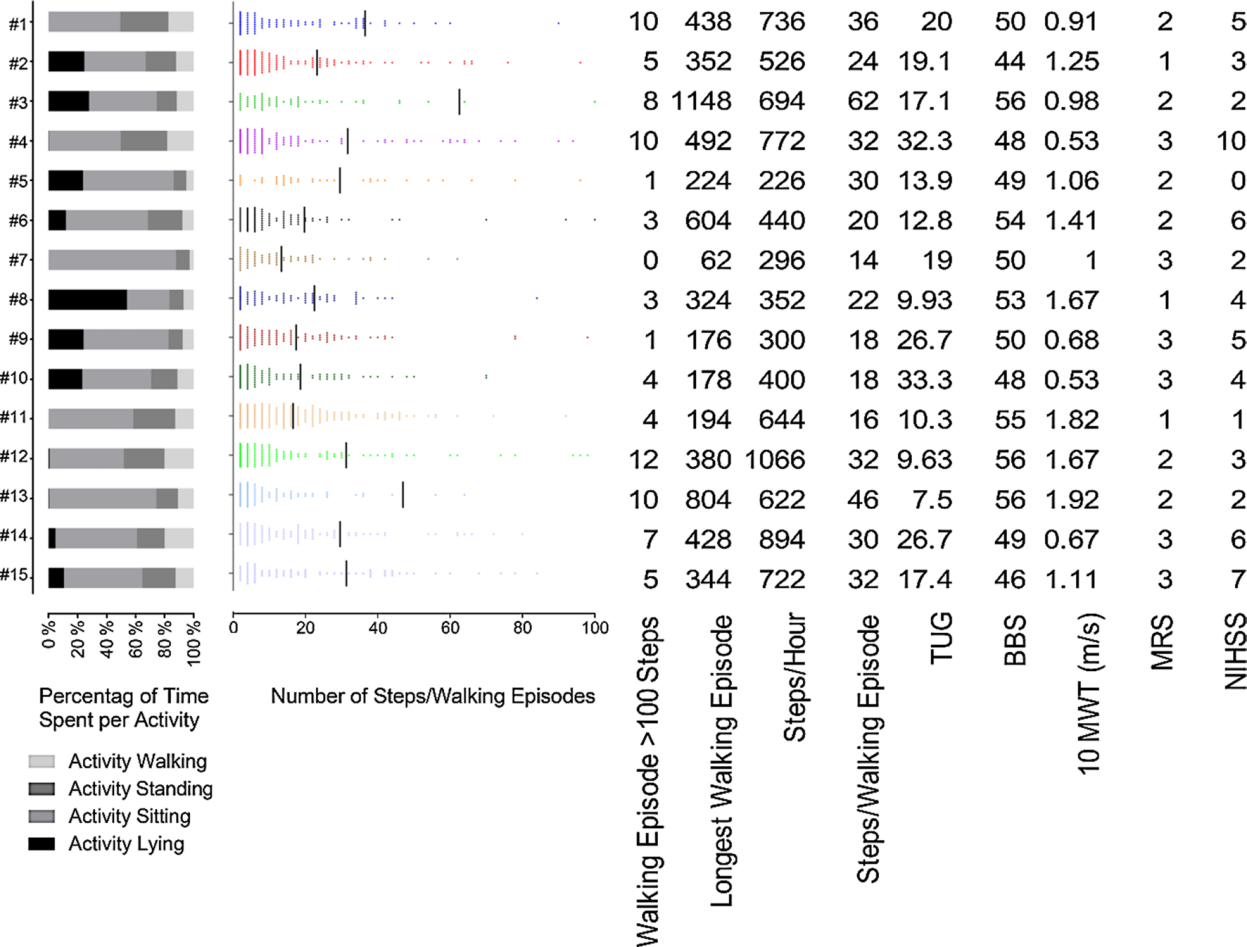
Summary of individual physical activity profiles, walking performance and clinical capacity measures. The stacked columns on the left depict individual physical activity profiles (lying, sitting, standing, and walking). In the plots in the middle section, all recorded walking episodes with up to 100 steps/episode are shown individually for all patients. Datapoints depict the number of steps. In the table on the right, sensor-derived measures of walking performance and clinical parameters of mobility (TUG), balance (BBS) and independence (MRS) are listed

To account for the varying length of recording time among different patients, the sensor-derived measures are reported as the percentage of time spent performing a given activity (e.g., walking) or as the number of activity units (e.g., steps) relative to the recording time. Specifically, the time spent walking, standing, sitting, and lying is reported as a percentage of the total recording time. Walking performance is reported as the average number of steps per recording hour (including time without ambulation) and as the average number of steps per walking episode. A walking episode was defined as an episode with at least 1 stride (2 consecutive steps) and a maximum of 6 s between two consecutive strides. The distribution of step numbers for walking episodes up to 100 steps is presented individually for each patient. The number of walking episodes with more than 100 steps and the number of steps of the longest walking episode are also reported in Fig. 2. Arm activity was quantified while sitting to prevent overestimation due to walking. The activity time of the impaired arm is reported in seconds per hour of time classified as sitting. The relative arm activity is the ratio of the activity time of the impaired arm to the activity time of the unimpaired arm while sitting.

The existing algorithms were developed in the framework of their respective research studies and implemented in MATLAB® (The MathWorks Inc., Version 7.10.0 (R2010a). Natick, Massachusetts USA), and data were postprocessed.

### Statistical analysis

Descriptive statistics were performed with GraphPad Prism® (GraphPad Software, Version 5.00, San Diego, California USA).

## Results

### Clinical measures of functional capacity

All clinical measures and patient-reported outcomes are listed in Table 2. All patients were living at home and were independent for most activities of daily living (ADL), as indicated by an average Barthel Index of 97.3. Three out of 15 patients had no significant disability (MRS = 1), 6 had slight disability, and 6 had moderate disability (MRS = 2 and 3, respectively). The NIHSS scores ranged from 0–10, indicating mild to severe stroke severity, with an average score of 4. None of the patients used a walking aid. The patients showed mild balance deficits, with Berg Balance Scale scores ranging from 44 to 56 and an average score of 50.9. The clinically measured walking speed (10 MWT) ranged from 0.5 to 1.9 m/s. The TUG results ranged from 7.5 to 33.3 s. The clinical assessments of upper limb movement showed a wide range of functional impairment, ranging from 9 (severe impairment) to 66 points (no clinical impairment) on the FMA of the upper limb and from 0–57 points on the ARAT. The individual values of clinical and performance data are reported in Fig. 2 and Table 3.

**Table 2:** Clinical measures of motor capacity, patient-reported measures of upper-limb usage and independence, and sensor-based measures of motor performance in daily life. Data are reported as the means ± standard deviation (minimum–maximum)

Clinical measures of capacity	
National Institutes of Health Stroke Scale (NIHSS)	4.0 ± 2.6 (0–10)
Action Research Arm Test (ARAT)	31.4 ± 24.0 (0–57)
Fugl-Meyer Assessment (impaired side)	40.3 ± 22.8 (9–66)
Timed Up and Go Test (TUG) 7 m (sec)	18.4 ± 8.2 (7.5–33.3)
10-Meter Walk Test (m/s)	1.1 ± 0.5 (0.5–1.9)
Berg Balance Scale (BBS)	50.9 ± 3.8 (44–56)
Patient report	
Motor Activity Log-14 (MAL-14, mean amount of use [AOU])	2.0 ± 1.5 (0.4–4.9)
Modified Ranking Scale (MRS)	2.2 ± 0.8 (1–3)
Barthel Index (BI)	97.3 ± 5.3 (85–100)
Sensor based measures of performance in daily life	
Time spent walking (%)	12 ± 5.3 (2.9–20.2)
Time spent standing (%)	19 ± 8.5 (8.7–33.2)
Time spent sitting (%)	55 ± 13.5 (29.3–87.8)
Time spent lying (%)	14 ± 15.6 (0.0–53.9)
Sedentary time (sitting or lying, %)	69
Steps/hour (of recording time)	579 ± 243 (226–1066)
Steps/walking episode	29 ± 13 (14–62)
Longest walking episodes (in steps)	410 ± 277 (62–1148)
Arm duration ratio (impaired/unimpaired) during sitting (%)	74 ± 20 (52–124)
Impaired arm duration (in sec) during sitting normalized per hour (sec/hour)	866 ± 341 (326–1570)

**Table 3:** Individual results of upper-limb motor capacity (FMA, ARAT), patient-reported arm usage (MAL-14, mean AOU) and daily life performance of arm activity during sitting (arm duration ratio and impaired arm duration)

	MAL-14 (mean AOU)	FMA	ARAT	Arm duration ratio (impaired/unimpaired) during sitting (%)	Impaired arm duration (in sec) during sitting normalized per hour (sec/hour)
Patient 1	1.33	15	9	66	518
Patient 2	1.15	11	0	58	594
Patient 3	1.5	51	41	73	1107
Patient 4	0.43	9	0	56	918
Patient 5	0.93	38	28	52	546
Patient 6	1.62	62	54	94	1570
Patient 7	4.79	58	57	124	326
Patient 8	4.86	65	57	70	883
Patient 9	0.64	33	15	52	643
Patient 10	2.64	66	57	86	1346
Patient 11	4.36	66	57	89	1102
Patient 12	1.79	55	45	94	1156
Patient 13	2.14	51	46	64	873
Patient 14	0.62	11	3	67	760
Patient 15	0.54	13	2	59	640

### Sensor-based measures of motor performance

The sensor-based measures of motor performance are summarized in the lower part of Table 2 as sample averages. In composite Fig. 2, the activity profiles of all patients are plotted as stacked columns on the left. All walking episodes up to 100 steps/episode are plotted as individual histograms in the middle part of Fig. 2. Further parameters of walking performance are listed alongside the clinical parameters on the right. Table 3 shows the individual values of patient-reported arm usage (MAL-14, mean AOU), clinical capacity measures of the upper limb (FMA, ARAT), and the sensor-based performance measures of arm activity in daily life.

## Discussion

The goal of this study was to examine the clinical value of information about motor performance in daily life in stroke patients for counseling and rehabilitation planning purposes. To do so, we focus on individual patients and discuss how information about motor performance can be used as a decision aid for the planning of individual rehabilitation programs or for counseling. The functional status of the 15 included patients was heterogeneous (Table 2), with NIHSS scores ranging from 0–10. The capacity measures of walking (walking speed between 0.5 and 1.9 m/s on the 10 MWT) and upper-limb functioning (ARAT scores between 0 and 57) also varied substantially among patients. We considered this heterogeneity in the patient population to be representative of a rehabilitation setting.

### Physical activity and walking

In line with previous research [31–33], the patients showed little physical activity on average, with a low fraction (12%) of time spent walking, and most of the time (69%) spent performing sedentary behaviors, i.e., lying or sitting (Table 2). The times spent walking and performing sedentary behaviors were highly variable among patients, irrespective of walking capacity (Fig. 2). The following patient examples demonstrate substantial discrepancies between the walking capacity and physical activity level in some patients. Patients #1, 4, 12, and 14 were rather active and spent 17–20% of their time walking. However, among these patients, only #12 was a comparably (i.e., compared to the other patients in this report) good walker according to the capacity tests (TUG: 10 s, 10 MWT: 1.7 m/s), whereas patients #4 and 14 were among the slowest walkers according to the capacity tests. On the other extreme were patients #5—9, who spent little time walking (5–8% of time) and were mostly sedentary (68–88% of time). Among these were patients with low as well as high walking capacities; patient #9 had a low walking capacity (TUG: 27 s, 10 MWT: 0.7 m/s), whereas patient #8 had a high walking capacity (TUG 10 s, 10 MWT 1.7 m/s).

Because a sedentary lifestyle is prevalent after stroke and constitutes an important vascular risk factor, counseling to increase daily physical activity is generally recommended for all stroke patients [34]. However, advocating a more elaborate program, such as a behavioral lifestyle interventions [35] to increase physical activity, may not be appropriate in patients who are active and meet the current recommendations, such as patients #1, 4, 12, and 14 (who spent 17–20% of their time walking). Information that a patient is mostly sedentary and spends little time walking may prompt the rehabilitation physician to enquire about the reasons, which may include low cardiorespiratory fitness, fear of falls, fatigue or depression [36, 37]. Based on the findings, he/she may specifically address the relevant issues, e.g., by developing a treatment plan focusing on cardiorespiratory fitness, reducing the risk of fall, or treating fatigue or depression.

These examples show that knowing a patient’s physical activity profile could aid the rehabilitation physician in choosing appropriate rehabilitative therapies. Additionally, not prescribing an unnecessary therapy, such as a behavioral lifestyle intervention [35], to increase physical activity in a patient who already meets the recommendations may be more economical than prescribing the same intervention to all patients.

Another important aspect of walking performance in daily life is the length of each walking episode. When asked whether walking faster or walking farther was more important, 76% of stroke patients reported that walking farther was more important in daily life because it allowed access to community facilities [38]. For older adults, a walking distance of 200–600 m was reported to be necessary for accomplishing community activities, such as going to grocery stores or banks [39]. In line with other studies [3, 6], the lengths of walking episodes in daily life were low in our study, with an average of 29 steps per walking episode and an average maximal walking distance per walking episode of 410 steps. The lengths of walking episodes varied substantially among patients, irrespective of their walking capacity (Fig. 2). For example, patients #3 and #7 both had average walking capacities, but they were at the opposite extremes regarding their walking episodes. Patient #3 had an average of 62 steps/episode, and their longest walk was 1148 steps (the highest values in this study), while patient #7 had an average of 14 steps per episode, and their longest walk was only 62 steps (the lowest values in this study). The situation of patient #7, who had only short walking episodes despite good walking capacity, may not be an exception in stroke patients. Lord et al. found that up to one-third of home-dwelling stroke patients do not walk unsupervised in their communities even though they have achieved good clinical mobility outcomes [40]. In patients with short average and maximal walking episodes (such as patient #7), an appropriate rehabilitation goal could be to increase the walking distance, with the intention of progressing from a household to a community walker and thus improving participation in daily activities.

On the other hand, some patients walked longer distances despite poor walking capacities. For example, patient #4 had one of the lowest walking capacities in this study (TUG: 32 s, 10 MWT: 0.53 m/s). However, in daily life, his walking performance was above average, with 32 steps per walking episode and a maximum walking episode of 492 steps. For this patient, longer walking episodes in daily life were not an issue, and increasing them would probably not be a priority. Instead, the rehabilitation goal could be to improve walking speed.

Counting daily steps with mobile devices has become a widespread practice in the general population. Numerous studies have explored the potential of counting daily steps to increase physical activity. A recent review and meta-analysis found that self-monitoring of step activity improved physical activity in patients with cardiovascular disease [41]. In this study, we did not report daily step counts due to the short recording time. Instead, the step activity is normalized to the duration of the sensor recording and reported as the number of steps taken per recording hour. The hourly step activity varied substantially among the patients in our study, irrespective of walking capacity. For example, patient #5 had the lowest step count per hour despite an average walking capacity (TUG: 14 s, 10 MWT: 1.06 m/s). The rehabilitation physician could discuss this low-step activity with the patient, identify barriers, educate the patient about the effects of physical activity, and set a realistic goal of increased step activity [42].

Although walking performance was not related to walking capacity in some patients, as shown by the examples above and in other studies [6, 40], capacity and performance correlated moderately in studies with larger samples. For example, walking capacity, measured with the 6 min walking test, correlated with walking performance (steps/day) in chronic stroke patients [9, 43, 44]. However, the clinical capacity measures explained only up to 54% of the variance in walking performance in these studies, suggesting that daily life performance cannot be inferred from clinical capacity measures in individual patients. Based on these findings, one could be misled to expect better performance in a patient with higher capacity than in a patient with lower capacity (and vice versa) and would not expect substantial discrepancies between capacity and performance, as reported in some patients in our case series and in other studies. However, the fact that at least 46% of the variance in walking performance could not be explained by capacity measures in these studies agrees with the finding of substantial discrepancies between capacity and performance in some patients. The cases discussed above showed discrepant walking capacity and performance measures, and only a moderate correlation was observed between the two in other studies [9, 43, 44], indicating that clinical assessments alone do not allow a complete picture of a patient’s walking status.

### Upper limb capacity

The clinical measures of arm capacity varied substantially among patients, ranging from severe to no clinical impairment (9 to 66 points on the FMA, average: 40.3 points, Table 3). As mentioned above, we considered this heterogeneity in the patient population to be representative of a rehabilitation setting. The total duration of impaired arm activity per hour of sitting ranged from 326 to 1570 s (≈ 5 to 26 min), with an average value of 866 s (≈ 14 min).

There were substantial discrepancies between clinical measures of arm capacity and sensor-derived measures of arm activity in some patients (Table 3). For example, among stroke patients with comparably good clinical capacity (FMA > 45 and ARAT > 40, i.e., patients #3, 6, 7, 8, 10, 11, 12, 13), there was a wide range in the absolute use of the impaired arm during sitting, ranging from 326 to 1570 s per hour of sitting time. Even patients #7, 8, 10, and 11, who all achieved the maximum score on the ARAT (i.e., 57 points), showed considerable variability in arm activity duration. For example, patient #10, who had 1346 s of normalized arm activity, had 4 times more arm activity than patient #7, who had 326 s. Patient reported arm use (MAL-14) in these four patients was above 4 points (= reportedly used the arm almost as much as they did before stroke), except in patient #10, with 2.6 points (= reportedly used the affected arm rarely or very rarely), who had the second longest arm activity of all patients. Discrepancies between capacity and performance measures of the upper limbs are well known [45–47] and can be explained by the concept of learned nonuse (i.e., failure to use the impaired arm despite adequate capacity) described by Taub and others [48, 49]. The recovery of capacity and performance in the upper limbs can diverge in some stroke patients, as shown in previous studies [5, 13], underscoring the need to assess both arm capacity and performance. Knowledge of low arm activity in daily life despite good arm function may prompt the rehabilitation physician to identify underlying reasons, such as learned nonuse of the affected arm. He/she may then plan specific interventions to increase arm usage in daily life, such as constraint-induced movement therapy [50].

## Limitations

The short motor performance recording time, at 5.03 h on average, may not be representative of individual patient performance in daily life. Previous research has suggested that 3 days of recording time would be optimal to obtain valid information on the daily activity of people with stroke [51]. We intended to monitor performance in a scenario that we considered feasible in a routine clinical setting. A longer monitoring time over several days would require the removal/reapplication or charging of sensors by patients or therapists, which was considered unrealistic in a routine clinical setting where time and resources are usually limited. An effort to reduce the number of sensors and the complexity of sensor handling while collecting relevant parameters and extending the recording time is necessary. We believe that quick setup and ease of use for both the patient and the physician or therapist is required for widespread use in clinical practice.

Furthermore, we cannot exclude that the clinical visit in the morning led to a bias because it potentially motivated the patients to be more active than usual. To minimize this potential bias, the patients were told to behave ‘as usual’.

## Conclusion

The discussed cases illustrate how information on motor performance in daily life in stroke patients can be used clinically to guide decisions regarding rehabilitation therapies and counseling. This information cannot be inferred from capacity measures, as demonstrated by the substantial discrepancy between capacity and performance in some patients. An array of information that includes clinical capacity measures, patient-reported outcomes and performance data should inform therapy decisions to tailor therapies to individual patients’ needs. It remains to be shown whether considering information about daily life motor performance for therapy planning translates into improved outcomes.

## Data Availability

The data that support the findings of this study are available from [F. Massé, K. Aminian, EPFL…]. However, restrictions apply to the availability of these data, which were used under license for the current study and thus are not publicly available. Data are, however, available from the authors upon reasonable request and with the permission of [F. Massé, K. Aminian, EPFL….]. This article should be cited as follows: Flury D, Massé F, Paraschiv-Ionescu A, Aminian K, Luft A, Gonzenbach R. Clinical value of assessing motor performance in postacute stroke patients.

## Abbreviations

NIHSS: National Institutes of Health Stroke Score
BI: Barthel Index
ARAT: Action Research Arm Test
FMA: Fugl-Meyer Assessment
TUG: Timed Up and Go Test
2 MWT: 2-Minute Walk Test
10 MWT: 10-Meter Walk Test
MRS: Modified Rankin Scale
MAL-14: Motor Activity Log-14
AOU: Amount of Use
BBS: Berg Balance Scale
ADL: Activities of daily living
